# TB incidence and characteristics in the remote gulf province of Papua New Guinea: a prospective study

**DOI:** 10.1186/1471-2334-14-93

**Published:** 2014-02-20

**Authors:** Gail B Cross, Katie Coles, Mandana Nikpour, Owen A Moore, Justin Denholm, Emma S McBryde, Damon P Eisen, Beverlyn Warigi, Robyn Carter, Sushil Pandey, Paul Harino, Peter Siba, Christopher Coulter, Ivo Mueller, Suparat Phuanukoonnon, Marc Pellegrini

**Affiliations:** 1 Infection and Immunity Division, Walter and Eliza Hall Institute of Medical Research, 1G Royal Parade, Parkville, VIC 3052, Australia; 2 Papua New Guinea Institute of Medical Research, Goroka, Eastern Highlands Province 441, Papua New Guinea; 3 Department of Rheumatology, St Vincent’s Hospital Melbourne, Fitzroy, VIC 3065, Australia; 4 Department of Medicine, St Vincent’s Hospital Melbourne, University of Melbourne, Fitzroy, VIC 3065, Australia; 5 Victorian Infectious Diseases Service, Royal Melbourne Hospital, Parkville, VIC 3050, Australia; 6 Barcelona Center for International Health Research, Barcelona, Spain; 7 Queensland Mycobacterium Reference Laboratory, Pathology Queensland, Royal Brisbane and Women’s Hospital Herston, Herston, QLD 4029, Australia; 8 Department of Medical Biology, University of Melbourne, Parkville, VIC 3010, Australia

**Keywords:** Papua New Guinea, Tuberculosis, Mycobacterium, Incidence, Drug resistance, MDR-TB, HIV, Kikori, GeneXpert

## Abstract

**Background:**

The incidence and characteristics of tuberculosis (TB) in remote areas of Papua New Guinea (PNG) are largely unknown. The purpose of our study was to determine the incidence of TB in the Gulf Province of PNG and describe disease characteristics, co-morbidities and drug resistance profiles that could impact on disease outcomes and transmission.

**Methods:**

Between March 2012 and June 2012, we prospectively collected data on 274 patients presenting to Kikori Hospital with a presumptive diagnosis of TB, and on hospital inpatients receiving TB treatment during the study period. Sputum was collected for microscopy, GeneXpert analysis, culture and genotyping of isolates.

**Results:**

We estimate the incidence of TB in Kikori to be 1290 per 100,000 people (95% CI 1140 to 1460) in 2012. The proportion of TB patients co-infected with HIV was 1.9%. Three of 32 TB cases tested were rifampicin resistant. Typing of nine isolates demonstrated allelic diversity and most were related to Beijing strains.

**Conclusions:**

The incidence of TB in Kikori is one of the highest in the world and it is not driven by HIV co-infection. The high incidence and the presence of rifampicin resistant warrant urgent attention to mitigate substantial morbidity in the region.

## Background

On a global scale, a substantial proportion of the morbidity and mortality caused by tuberculosis (TB) is attributed to HIV co-infection and the emergence of drug resistance [1]. These factors and their impact on TB incidence and outcomes have been well studied in many African countries [2-4]. The incidence of TB in countries with geographically isolated communities such as Papua New Guinea (PNG) is largely unknown and there is very limited data on drug resistance and HIV co-infection in remote PNG communities.

The World Health Organization (WHO) reported annual national incidence of TB in PNG is 346 per 100,000 population and the reported percentage of tested TB patients that are HIV positive is 11% [5]. Due to the geographical isolation of most rural areas and limited access to health care facilities in these areas, the national figure may not reflect true TB incidence in the many remote regions of PNG. It is important to quantify TB incidence in these areas as many resource development projects are opening access to these remote communities and modeling studies performed in the Western province of PNG (adjacent to the Gulf province) have indicated that areas with high disease burden can dominate the TB dynamics of an entire region [6]. Resource development ventures with projects extending into the Gulf province have raised concerns that TB is prevalent in the area and this was brought to our attention. Therefore, we sought to determine the incidence of TB, the frequency of drug resistance and the incidence of concurrent HIV infection in the remote Kikori community of PNG.

## Methods

### Setting

Kikori is a town located in the Kikori district of the Gulf province of Papua New Guinea (Additional file 1: Figure S1). The district is divided into 4 rural local level governments (LLG): West Kikori LLG, East Kikori LLG, Baimaru LLG and Ihu LLG. Kikori district encompasses an area of 27000 km^2^, only 2500 km^2^ of which is inhabited by a network of villages scattered along a river delta system [7]. There are two hospitals in the Kikori district. Kikori hospital primarily services West Kikori and East Kikori LLGs and a few patients from Baimaru LLG attend the hospital. Kikori hospital is a 90-bed hospital administered and operated by the Christian charity organization called Gulf Christian Services. There are 25 medical staff at Kikori hospital and volunteer expatriate physicians sporadically visit the hospital. Over the study period, two Australian physicians attended the hospital to assist with clinical care and data collection. Kikori hospital resident staff managed TB diagnosis, treatment and care. Standard care for TB patients at Kikori generally involved hospital admission, where possible, for the first 2-months of intensive phase treatment followed by 4 months of treatment at home. Hospitalization provides a means of directly observing therapy and monitoring for side effects. TB cases are segregated/isolated in a designated TB ward that is physically separated from all other hospital wards. TB patients are provided with hospital appointments, if they reside near the hospital, or they are referred to local health facilities for treatment follow-up. Patients residing in very remote areas are provided with a full treatment course to be completed at home if alternative follow-up arrangements are impractical. DOTS intervention had not been implemented at Kikori during the study period. The Kikori district health office TB control program was responsible for TB patient follow-up, but due to funding and manpower issues the program was not functioning. Accessibility to Kikori town and Kikori hospital is poor.

### Study design

Between March 2012 and June 2012, we conducted an observational study to determine the incidence of TB and disease characteristics in Kikori. Patients self-presenting to Kikori District Hospital with TB symptoms and inpatients receiving TB treatment during the study period were invited to participate in the study. Written informed consent was obtained prior to study enrolment. The PNG \Medical Research Advisory Committee approved the study (amendment to MRAC No. 10.17) and maintains records of all signed consent forms. All patients attending Kikori hospital seeking medical attention were triaged as per hospital policy by medical workers. Patients with symptoms or signs suggestive of TB, including fever, unexplained weight loss, cough, breathlessness, lymphadenopathy, abdominal pain, abdominal distention and neurological symptoms or signs were then referred to an infectious diseases physician. The specialist physician independently interviewed patients and performed a medical examination. Patients with a medical history and examination that was consistent with TB were enrolled into the study as TB suspects. A diagnosis of TB was made by an infectious diseases physician based on the criteria listed below (Diagnosis of TB) and TB suspects confirmed to have TB were reclassified as TB cases. All patients prospectively diagnosed, as TB cases, were included in our estimation of TB incidence.

### Diagnosis of TB

Three sputum samples were collected from patients with a productive cough. Ziehl-Neelsen (ZN) stains were performed on sputum and other clinical specimens by trained scientists. Aliquots of sputum specimens were frozen and stored at −20°C for later analysis as described below. Attempts were made to obtain other clinical specimens for ZN smears; this included aspirates from lymph nodes, swabs from discharging ears and aspirates from other collections of pus. The WHO recommended algorithm for the diagnosis of TB, incorporating the international standards for TB care, were used for the diagnosis of TB in this study [8].

### Definition of TB cases and new and previously treated cases

The patients enrolled were classified as TB suspects or cases of TB in accordance with the WHO TB Treatment guidelines [9]. A ‘TB suspect’ was defined as a patient with symptoms or signs of pulmonary or extra pulmonary TB and/or constitutional symptoms (loss of appetite, weight loss, fever and fatigue). A ‘TB case’ was defined as a patient with acid fast bacilli (AFB) detected in ZN stained sputum (or other) specimen, or in the absence of such laboratory confirmation, when an infectious diseases physician diagnosed TB based on symptoms and signs and decided to treat the patient with a full course of TB therapy. Cases of TB were further classified as new or previously treated cases [9].

### Estimation of population size and TB incidence

Detailed population figures for each province in PNG are available from the 2000 PNG National census. The population serviced by Kikori Hospital in 2000 was 17,779 people. This is the sum of the population in West Kikori rural ward (n = 7579), East Kikori rural ward (n = 8788) and people residing in Baimaru Station in Baimaru rural ward (n = 1416). The annual population growth rate from the most recent census in 2011 is known to be 1.2% (for the Gulf province), however absolute population figures have not yet been made available. Therefore the population serviced by Kikori Hospital in 2012 is estimated to be 20,144 people. We validated the catchment area detailed above by confirming that all obstetrics patients attending the hospital over the study period resided in the catchment area and that all TB cases in a retrospective review of hospital records (2004–2011) resided in the defined catchment area.

### HIV testing

HIV testing was performed using the Alere Determine HIV-1/2 Ag/Ab Combo Rapid test kit HIV 1/2 STAT-PAK, Chembio Diagnostic Systems (NY, USA), after patients received appropriate counseling.

### GeneXpert testing, microscopy culture and genotyping

Each sputum sample was analyzed by two trained microscopists for AFB at Kikori Hospital. Aliquots of all sputum samples obtained from cases were frozen and stored at −20°C for several months before being sent to the PNG Institute of Medical Research (Madang) or the Queensland Mycobacterial Reference Laboratory (MLR) in Australia for GeneXpert assays. Sputum samples were decontaminated according to Petroff’s method [10] and GeneXpert assays utilizing the Xpert MTB/RIF kit (Cepheid, Sunnyvale California USA) were performed as described previously [11]. Additionally, Queensland MLR cultured all AFB smear positive sputa using BACTEC™MGIT™ (Beckton Dickinson, USA) and performed drug susceptibility testing (DST) and genotyping analyses on all isolates that were recovered after culture. DST was performed by the proportion method [12] using BACTEC™MGIT™960 [13]. DNA was purified from cultures using ethanol and heating to 95°C and multilocus mycobacterial interspersed repetitive unit variable number of tandem repeats (MIRU-VNTR) was used for molecular analysis and to define strains as previously described using GenoScreen (Lille Cedex, France) MIRU-VNTR Typing Kit – *Mycobacterium tuberculosis* Complex 24-loci [14]. Amplified products were detected using the AB® 3130xl genetic analyzer with the GeneScan™ 1200 LIZ® size standard (Appliedbiosystems, Life Technologies, NY, USA). The results were analyzed using GeneMapper® Software (Appliedbiosystems, Life Technologies, NY, USA). The online MIRU-VNTRplus tool (http://www.miru-vntrplus.org) was used to calculate a neighbor-joining tree using categorical distance measures based on 24 loci MIRU-VNTR, and to represent strains and lineage similarities as previously described [15,16]. The loci (and corresponding aliases) used in our studies were: 154 MIRU02, 424 Mtub04, 577 ETRC, 580 MIRU04 ETRD, 802 MIRU40, 960 MIRU10, 1644 MIRU16, 1955 Mtub21, 2059 MIRU20, 2163b QUB11b, 2165 ETRA, 2347 Mtub29, 2401 Mtub30, 2461 ETRB, 2531 MIRU23, 2687 MIRU24, 2996 MIRU26, 3007 MIRU27 QUB5, 3171 Mtub34, 3192 MIRU31 ETRE, 3690 Mtub 39, 4052 QUB26, 4156 QUB4156 and 4348 MIRU39.

### Data collection

Data were collected through patient interview for all TB cases, and stored in a dedicated database.

### Demographic variables

The following demographic variables were recorded for each patient: age, gender, body mass index (BMI), occupation, level of education, access to mobile phone, distance from hospital, smoking status, alcohol consumption, exposure to in-house smoke and chewing of betel nut. BMI for children and teenagers was calculated using the Centers for Disease Control and Prevention BMI online calculator tool (http://apps.nccd.cdc.gov/dnpabmi/). Exposure to smoke from cooking fires has been associated with an increased risk of TB [17], and spitting associated with betel nut chewing may facilitate transmission of infection in crowded housing [18]. Another variable captured was ethnicity (Papuans and Highlanders), to ascertain the ethic representation amongst TB cases. Patients receiving TB therapy but diagnosed prior to the study commencement were included in the description of TB characteristics and demographics. These inpatients were specifically receiving treatment for TB as part of their standard hospitalized intensification phase of treatment.

### Risk factors for TB

The following potential risk factors for TB were recorded for each patient: contact with a TB case, history of previous TB treatment, crowding (number of people in cohabitation), and absence of a BCG vaccination scar.

### Distribution of TB cases

Global Positioning System (GPS) coordinates for the homes and villages of 143 TB cases were ascertained by visiting the villages and using a Garmin eTrex 10 GPS device. GPS coordinates were plotted using Epi Info 7 software (Center for Disease Control, USA).

### Time trends in TB incidence in Kikori

Hospital records of the National TB Register from 2004 to 2012 were reviewed to obtain the number of patients commenced on TB therapy every year.

### Statistical analysis

Confidence intervals for TB incidence were calculated using both the Wilson score interval and Agresti-Coull adjusted Wald interval methods.

## Results

### Patient characteristics

**Figure 1:**
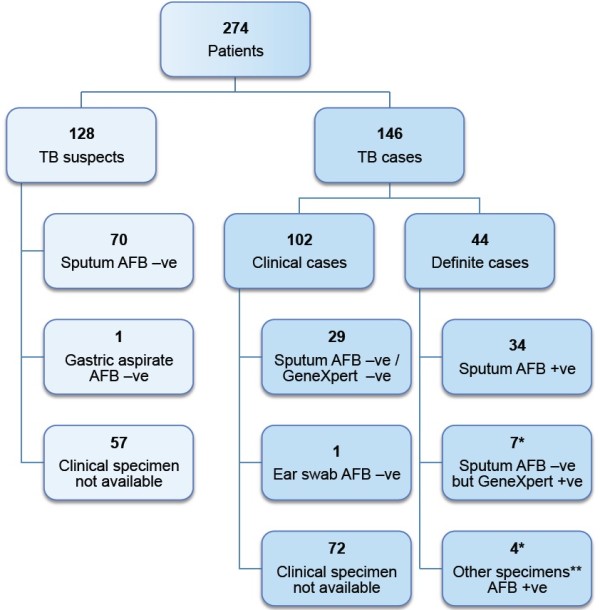
**Number of patients enrolled in the study, diagnostic results and case categories.** *One case had an AFB –ve but GeneXpert + ve sputum along with an AFB + ve lymph node aspirate. **Other clinical specimens included: two lymph node aspirates, one ear swab and one abscess aspirate.

Two hundred and eighty seven people were approached for informed consent. Thirteen declined consent and 274 were enrolled into the study. Of the 274 patients enrolled; 146 were determined to be TB cases and 128 were TB suspects (Figure 1). Forty nine of the 146 cases were inpatients receiving TB treatment as part of their intensification phase of therapy and the remaining 97 were prospectively diagnosed TB cases accrued over the study period. Clinical specimens were available for analysis in 56 out of the 97 cases. In the remaining 41 cases we were unable to collect adequate specimens for analysis. Sputum and clinical specimens from 39 of the 56 cases were AFB positive or had MTB DNA detected by GeneXpert (Table 1). The demographic characteristics of all TB cases are detailed in Table 2.

**Table 1:** Specimen results for 97 prospectively accrued cases

**Clinical specimen**	**Total number**	**Microscopy (numbers)**		**Xpert MTB/RIF (numbers)#**	
**New + relaspe cases (N=80)**					
No clinical specimens	33				
Sputum	45	AFB smear negative (18)		MTB DNA detected (5/16**)	
				High	(0)
				Medium	(0)
				Low	(2)
				Very low	(3)
				***rpOB *** **mutation**	(0)
		AFB smear positive (27)		MTB DNA detected (25/26*)	
		Scanty	(2)	High	(11)
		1+	(2)	Medium	(6)
		2+	(11)	Low	(6)
		3+	(12)	Very low	(2)
Back abscess aspirate	1			***rpOB***	(2)
Ear swab	1	1+			
		2+			
**Treatment failure/defaulted cases (N=17)**					
No clinical specimens	8				
Sputum	8				
		AFB smear negative	(4)	MTB DNA detected	(0/3*)
		AFB smear positive	(4)	MTB DNA detected	(2/3*)
		Scanty	(1)	High	(2)
		1+	(0)	Medium	(0)
		2+	(0)	Low	(0)
		3+	(3)	Very low	(0)
Lymph node aspirate	1	1+		***rpOB *** **mutation**	(1)

#Non-sputum specimens were not analyzed by Xpert.

Lost sputum specimens could not be analyzed with Xpert (*1 lost, **2 lost).

**Table 2:** Demographic characteristics of 146 TB cases

**Characteristic**	**n (%)**
**Median age**	22 (range: 8 months to 76 years)
**Age**	
< 5 years old	25 (17%)
5 – 15 years old	27 (18%)
16 – 59 years old	90 (62%)
≥ 60 years old	4 (3%)
**Sex**	
Male	66 (45%)
Female	80 (55%)
**Ethnicity**	
Papuan	141 (97%)
Highlanders	5 (3%)
**Occupation**	
Subsistence farmers	65 (44%)
Students	41 (28%)
Mining industry	15 (10%)
Health workers	3 (2%)
Children/unknown	40 (27%)
**Level of education**	
University education	1 (0.7%)
Diploma	2 (1.4%)
Trade school	2 (1.4%)
High school education	23 (15.8%)
Primary school education	51 (34.9%)
No formal education	33 (22.6%)
Unknown/children under school age	34 (23.2%)
**Mobile phone access**	
NO access to mobile phone/no coverage	55 (37.7%)
Access to own mobile phone	51 (34.9%)
Access to family members mobile phone	40 (27.4%)
**Distance from hospital**	
Walk ≤ 1 hour	46 (31.5%)
Boat road trip ≤ 1 hour	29 (20%)
Walk > 1 hour < 1 day	0
Boat road trip > 1 hour < 1 day	44 (30%)
Walk ≥ 1 day	5 (3.4%)
Boat/road trip ≥ 1 day	22 (15.1%)
Smoking status*	
Non smokers	104 (71.2%)
Smokers	41 (28.1%)
Unknown	1 (0.7%)
Exposure to in-house smoke**	
Exposed	104 (71.2%)
No exposure	32 (21.9%)
Unknown	10 (6.9%)
**Alcohol consumption**	
Drink alcohol	30 (20.5%)
Never drink alcohol	115 (78.8%)
Unknown	1 (0.7%)
**Betel nut chewing**	
Does not chew betel nut	92 (63%)
Chews betel nut	53 (36.3%)
Unknown	1 (0.7%)

*Actively smoking any quantity of cigarettes prior to and leading up to illness.

**Any exposure to wood smoke from in-house fire prior to and leading up to illness.

The median age of TB cases was 22 years and 36% of all TB cases were under the age of 16 years. Fifty eight percent of cases had had no formal education or had only completed primary school education. Of note, 62% of cases had access to functional mobile phones. Twenty percent of patients had to travel for more than one day to get to the hospital. Most patients were non-smokers but 71% had exposure to in-house smoke from cooking fires. Potential risk factors for TB in cases are detailed in Table 3. Seventy three percent of cases reported having had had contact, usually through co-habitation, with at least one other person that was diagnosed with TB. Many households consisted of more than 10 occupants in a single dwelling.

**Table 3:** Potential risk factors for TB in 146 TB patients

**Risk factor**	**Frequency**
Contact with TB case	107 (73.3%)
No contact with TB	34 (23.3%)
Unknown	5 (3.4%)
Number of people in a household	
≤ 5	35 (24.0%)
6 – 10	71 (48.6%)
> 10	40 (27.4%)
No history of TB	99 (67.8%)
Unknown	1 (0.7%)
History of previously treated TB	46 (31.5%)
Relapsed case	11/46 (23.9%)
Treatment failure	6/46 (13.1%)
Defaulted on therapy	18/46 (39.1%)
Completed treatment but uncertain if symptoms resolved	11/46 (23.9%)
BCG scar present	99 (67.8%)
No BCG scar	41 (28.1%)
Unknown	6 (4.1%)

### Microbiology

All sputum and clinical specimens, obtained from cases, were ZN-stained and the number of AFB was quantified (Table 1). Eighty one percent of AFB positive sputa (25/31) had a heavy burden of bacilli (2+ or 3+). We were able to analyze 48 out of 53 sputum samples (5 were lost) with GeneXpert for the prescence of *Mycobaterium tuberculsosis* (MTB) DNA and rifampicin resistance (*rpoB* gene mutation) [10,11]. Twenty seven of the available 29 smear positive sputa had MTB DNA detected by GeneXpert and three of these had rifampicin resistance detected. MTB DNA was also detected in 5 out of the available 19 AFB smear negative specimens obtained from TB cases, and none of these were rifampicin resistant on GeneXpert analysis (Table 1). There was a wide geographical distribution of TB cases and rifampicin resistant isolates across the region (Additional file 1: Figure S2). One out of the three patients with rifampicin resistance had previously been treated for TB and had defaulted on therapy. The other 2 patients had no history of TB treatment.

**Figure 2:**
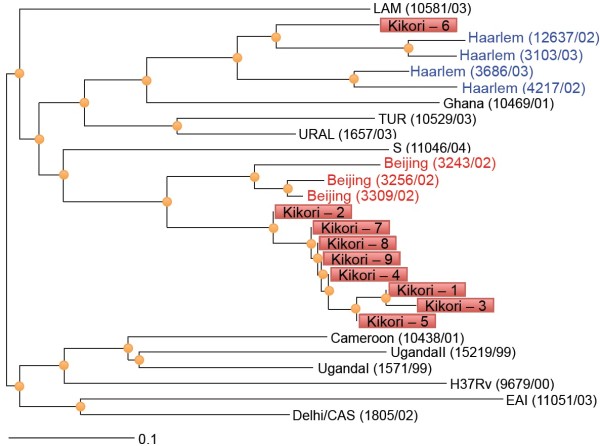
**Phylogentic tree of 9 cultured isolates from Kikori.** Numbers in parentheses represent MIRU-VNTR*plus* database strain ID numbers.

All sputum specimens that were AFB positive were cultured and 10 specimens yielded isolates. DST confirmed that all of the cultured isolates were sensitive to first line drugs. Genotypic profiling was performed on these isolates using MIRU-VNTR. A complete pattern was only available for 9 isolates from cases distributed across the Kikori region (Additional file 1: Figure S3). Most isolates resembled Beijing strains and allelic diversity was noted at two loci (Figure 2).

### TB clinical manifestations and treatment follow-up

A complete summary of clinical symptoms and signs for TB cases is shown in Additional file 1: Table S1. Thirty four percent of children were severely underweight with BMIs falling below the 1^st^ percentile. The clinical focus or site of TB in cases is shown in Table 4. We next assessed factors that may be contributing to the development of TB and impact its management. HIV screening was performed on 105 TB cases. We identified 2 TB cases that were co-infected with HIV (1.9%). We collected data on patient management, discharge and follow-up. By the end of the study, 37 of 146 cases (25%) were inpatients, 17 (12%) were being managed as outpatients and 7 (5%) were transferred to another hospital closer to their home village or to mining camp clinics if employed by the mining sector. Sixteen cases (11%) defaulted on their therapy during the course of the study with the most common reason being a lack of food provision at the hospital (7/16). The remaining cases (46%) were discharged to their home with a supply of TB medication because of hospital bed shortages. Two cases (1.4%) died during the study period but formal mortality rates could not be calculated, as many cases were lost to follow-up after hospital discharge. Only 24 of 83 cases (29%) that were discharged from hospital, with TB therapy, attended any follow-up appointment.

**Table 4:** TB in 146 cases by site

**TB site**	**No. of patients**	**Positive ZN smear**	**Median age (age range)**
Disseminated TB	14 (9.6%)	1 sputum	7 (1 – 35)
		1 ear swab	
		1 lymph node aspirate	
Pulmonary TB	72 (49.3%)	34 sputa	30 (< 1 – 76)
		7 smear negative but	
		MTB DNA detected	
TB Lymphadenitis	28 (19.2%)	1 aspirate	10 (< 1 – 40)
TB Abdomen/Pelvis	11 (7.5%)	-	19 (2 – 45)
Cerebral TB/TB Meningitis	6 (4.1%)	-	4 (2 – 5)
TB malnutrition	6 (4.1%)	-	3.5 (< 1 – 5)
TB spine	3 (2.1%)	1 aspirate	25 (8 – 47)
TB in Joint	1 (0.7%)	-	6
Site unknown	5 (3.4%)	-	28 (24 – 56)

### Incidence of TB in Kikori

**Figure 3:**
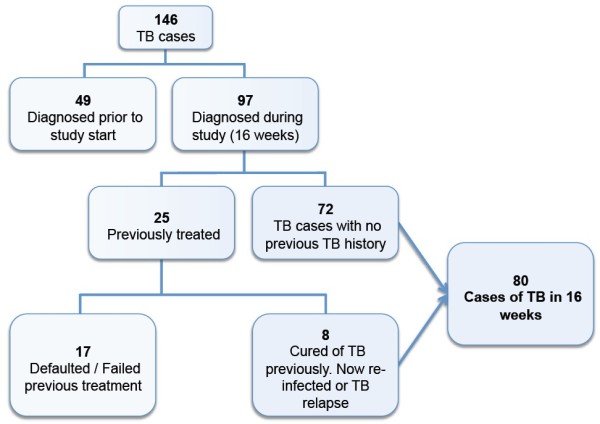
Flow chart for calculating incidence of TB in Kikori.

Over the course of the study period the number of TB cases enrolled per month remained consistent, ranging from 20 to 33 per month. To estimate TB incidence we only included cases that were prospectively diagnosed and commenced on treatment during the study period (n = 97). Based on the number of new, relapse and re-infection cases (Figure 3) over 16 weeks, we extrapolate the TB incidence to be 1,290 cases per 100,000 people, per year (95% CI: 1140 to 1460 per 100,000) in the Kikori region (West and East Kikori LLGs and Baimaru Station in the Baimaru LLG). The incidence of bacteriologically confirmed TB was 550 cases per 100,000 people, per year. We next assessed if the large number of TB cases was a recent or more chronic problem. We reviewed hospital records to track the number of registered TB cases at Kikori Hospital between 2004 – 2011 (Additional file 1: Figure S4). The number of expected cases for 2012 based on extrapolated figures from our 16-week study is 315, and this is consistent with figures over the preceding 8 years.

## Discussion

We have identified one of the highest TB incidence rates in the world [5], much higher than the WHO figure reported in 2011 [19]. We used the same case definitions in our study that are used by the PNG government in reporting National incidence to WHO. Our incidence figure may be an underestimate of the true incidence, as only patients presenting to the hospital were included in the study and we did not perform active case finding in the community villages. Equally, however, the inability to bacteriologically confirm all TB cases, due to the resource poor setting, may have led to an over diagnosis of TB. The incidence we report is not a temporally isolated event coincident with our study as a review of Kikori Hospital TB registries indicates that TB has been problematic in the area since at least 2004 (Additional file 1: Figure S4). We used the best available tools to estimate the population in the region but migration out of the region, variations in population growth and inaccuracies in census data may have led to under or over estimates of the population size used to calculate TB incidence. In contrast to the high TB incidence rates reported in African countries [20], the rate in Kikori is unusual as it is not associated with a high frequency of HIV co-infection. Several factors are probably contributing to the high incidence of TB in Kikori including: delayed presentation, difficulties in managing patient follow-up, infectious burden, and the interplay between local environmental factors and immunity. Our analysis of case demographics, disease characteristics and microbial genetics sheds some light on the likely factors that contribute to the Kikori TB incidence.

Delayed presentation is evident in our study as illustrated by the number of complicated presentations including disseminated TB, and the duration of symptoms. We found literacy to be poor and in the absence of education, cultural beliefs and folklore can delay the seeking of early treatment for TB [21]. Additionally the remoteness of some villages precluded early access to medical care and compromised treatment and follow-up. Access to medical care and follow-up could potentially be improved by contacting patients that return home using mobile phone. The remoteness of the hospital from major drug distribution centers also complicated supply of both loose and fixed combination anti-TB drugs. The late presentation of cases and the inefficiencies in treatment access and delivery may contribute to prolonged transmission risk and very high infectious burdens of MTB. Factors such as overcrowding, spitting as a cultural phenomenon, and the heavy AFB density in most cases, could contribute to a high infectious load which may lead to cycles of infection, repeated exposure, onset of clinical disease, and further transmission of disease. It is unclear what the major drivers of clinical disease in Kikori are but factors that may contribute to disease development include malnutrition and exposure to household smoke [22,23].

Our ability to culture specimens and obtain isolates for genotyping and other analyses was compromised by difficulties in maintaining a cold chain during storage and transport. Culture recovery rates in the Australian reference laboratory were low, although the Xpert MTB/RIF assay performed well on smear positive samples despite the loss of viability. Of the ten isolates grown and drug susceptibility completed, all were pan susceptible to the five first line agents, including pyrazinamide. We were not able to recover culture isolates from the three sputa that were found to harbour *rpoB* mutations. While rifampicin resistance is considered a strong predictor of multidrug resistant phenotype [24,25], the association is not invariable [26] and cannot necessarily be inferred in Kikori where no prior knowledge of drug resistance patterns exist. Although sample size is small, the finding of three rifampicin resistant isolates amongst 32 strains tested (9%) is of concern and a systematic study of patterns of drug resistance to anti-tuberculous agents is urgently required in this patient cohort.

Whilst the exact factors driving TB in Kikori are unclear, it would appear that circumstances could easily conspire to create an environment for the generation and spread of MDRTB. This is a major concern for a region that has limited access to, and ability to deliver and monitor second line therapies. In the absence of substantial improvements in infrastructure and supports, ad hoc administration of second line therapy could result in the development of extensively drug resistant TB [27]. The high TB incidence in Kikori could rapidly promote the dominance of resistant TB strains unless a reliable TB control program is implemented that can detect cases, support microbiological diagnosis including rapid detection methods such as Xpert MTB/RIF and ensure treatment adherence and follow-up. The more immediate consequences of the high incidence of TB in Kikori and its impact on morbidity and mortality need to be urgently addressed.

## Conclusion

Our study shows that, in at least one remote region in PNG, there is an extremely high incidence of TB, with the potential to promote the emergence and expansion of MDRTB. The extension of commercial resource development projects into remote areas of PNG has helped identify the problem but it may also serve to disseminate it beyond these remote communities.

## Abbreviations

AFB: Acid fast bacilli; BMI: Body mass index; HIV: Human immunodeficiency virus; MDRTB: Multidrug resistant tuberculosis; MTB: *Mycobacterium tuberculosis*; PNG: Papua New Guinea; TB: Tuberculosis; WHO: World Health Organization; ZN: Ziehl-Neelsen.

## Competing interests

None of the authors have any financial interests in this ExxonMobil PNG LNG and the study was conducted independently of ExxonMobil.

## Authors’ contributions

MP, SP, PS, IV proposed the initial idea for the study. MP, SP, GBC, MN contributed to the study design. GBC, KC, BW collected all the data. OAM, MN, MP, GBC assisted with database design and set-up. GBC, KC, SP, PH, PS, MP managed the project. GBC, DPE, SP, PH, PS, MP supervised staff. GBC, CC, RC, SP performed and or supervised microbial assays (excluding DST), GBC, MN, MP analyzed and interpreted the data. All authors contributed to the writing of the manuscript. All authors read and approved the final manuscript.

## Pre-publication history

The pre-publication history for this paper can be accessed here:

http://www.biomedcentral.com/1471-2334/14/93/prepub

## Supplementary Material

Additional file 1: Table S1 Clinical signs and symptoms of 146 TB patients. **Figure S1.** Representation of PNG with the Gulf province highlighted in red and expanded below to show some of the rural wards relevant to the study. Not all PNG islands and provinces are shown. Map not to scale. **Figure S2.** A map of the Gulf Province showing the distribution of 143 cases (yellow). Bottom map is an enlargement of the indicated area showing rifampicin resistant cases (red). **Figure S3.** Geographical distribution of six (of 9) genotyped isolates. **Figure S4.** Number of TB cases in Kikori between 2004 and 2011. Click here for file
