# Supplementary material for: TB incidence and characteristics in the remote gulf province of Papua New Guinea: a prospective study
Source: BMC Infect Dis. 2014 Feb 20;14:93. doi: 10.1186/1471-2334-14-93 (PMC3936911; doi:10.1186/1471-2334-14-93)
Supplement: Additional file 1: Table S1 — Clinical signs and symptoms of 146 TB patients. Figure S1. Representation of PNG with the Gulf province highlighted in red and expanded below to show some of the rural wards relevant to the study. Not all PNG islands and provinces are shown. Map not to scale. Figure S2. A map of the Gulf Province showing the distribution of 143 cases (yellow). Bottom map is an enlargement of the indicated area showing rifampicin resistant cases (red). Figure S3. Geographical distribution of six (of 9) genotyped isolates. Figure S4. Number of TB cases in Kikori between 2004 and 2011. [file 1471-2334-14-93-S1.doc]

**SUPPLEMENTARY DATA**

**Table S1: Clinical signs and symptoms of 146 TB patients**

| **Symptoms / Signs Frequency / Value** | |
| --- | --- |
| **Constitutional Symptoms** | |
| No Loss of weight (LOW)  Some LOW (1 – 5kg)  Pronounced LOW (> 5kg)  Loss of appetite  Fevers  Night sweats  **Other features**  Clinical anaemia  Median Hb (Haemocue, N = 69)**  Median BMI for adults (20 years and older)  Children (2 – 19 years) with BMI calculated  BMI at less than 1^th^ percentile (severely underweight)  BMI at less than 5^th^ percentile (underweight) | 20 (13.7%)  51 (34.9%)  75 (51.4%)  78 (53.4%)  96 (65.8%)  92 (63%)  78 (53.4%)  102 (26 – 156) g/L  19.2 (14.8 – 30.5) Kg/m^2^  41  14 (34.1%)  21 (51.2%) |
| **Respiratory Signs and Symptoms** | |
| Cough  1 – 2 weeks  > 2 weeks  Productive  Pleuritic chest pain  Haemoptysis  Lung pathology on auscultation  Unilateral  Bilateral  Signs of Pleural Effusion  Unilateral  Bilateral | 3 (63.7%)  12  81  66  62 (42.5%)  0  80 (55.2%)  38  42  18 (12.4%)  16  2 |
| **Extra-pulmonary Signs and Symptoms** | |
| Lymphadenitis on examination  Matted nodes  Abdominal mass  Abdominal tenderness  Abdominal mass + tenderness  Ascites  Severe headache + seizures or altered conscious state  Hemiparesis | 49 (33.6%)  15 (10%)  15 (10.3%)  35 (24.0%)  14 (9.6%)  4 (2.8%)  18 (12.4%)  5 (3.4%) |
| **Other Co-Morbidities**  Palpable liver or spleen  Random blood glucose > 10 mmol/L | 23 (15.8%)  1 (0.07%) |

**Figure S1:** Representation of PNG with the Gulf province highlighted in red and expanded below to show some of the rural wards relevant to the study. Not all PNG islands and provinces are shown. Map not to scale.

**Figure S2:** A map of the Gulf Province showing the distribution of 143 cases (yellow). Bottom map is an enlargement of the indicated area showing rifampicin resistant cases (red).

**Figure S3:** Geographical distribution of six (of 9) genotyped isolates.

**Figure S4:** Number of TB cases in Kikori between 2004 and 2011.
